# Assessing the Potential of Inoculative Field Releases of *Telenomus remus* to Control *Spodoptera frugiperda* in Ghana

**DOI:** 10.3390/insects12080665

**Published:** 2021-07-22

**Authors:** Lakpo Koku Agboyi, Babatoundé Ferdinand Rodolphe Layodé, Ken Okwae Fening, Patrick Beseh, Victor Attuquaye Clottey, Roger Day, Marc Kenis, Dirk Babendreier

**Affiliations:** 1 CABI, Cantonments, Accra P.O. Box CT 8630, Ghana; v.clottey@cabi.org; 2 African Regional Postgraduate Programme in Insect Science (ARPPIS), University of Ghana, Legon, Accra P.O. Box LG 68, Ghana; ferdinandlayode76@yahoo.com (B.F.R.L.); kenof2@yahoo.com (K.O.F.); 3 Soil and Irrigation Research Centre (SIREC), School of Agriculture, College of Basic and Applied Sciences, University of Ghana, Legon, Accra P.O. Box LG 68, Ghana; 4 Plant Protection and Regulatory Services Directorate (PPRSD), Accra P.O. Box M 37, Ghana; pkbeseh@gmail.com; 5 CABI, 673 Limuru Road, Muthaiga, P.O. Box 633, Nairobi 00621, Kenya; r.day@cabi.org; 6 CABI, 1 Rue des Grillons, 2800 Delémont, Switzerland; m.kenis@cabi.org (M.K.); d.babendreier@cabi.org (D.B.)

**Keywords:** fall armyworm, maize, egg parasitoid, parasitism, damage, Ghana

## Abstract

**Simple Summary:**

The fall armyworm (FAW) is a serious threat to maize production in Africa following its outbreak in 2016. Fortunately, some beneficial insects that could control FAW are already present in Africa, including *Telenomus remus* which parasitizes FAW eggs. *Telenomus remus* has been used in South America for biological control of FAW for several decades. Though *T. remus* is already present in Africa, little is known about its potential to be used for augmentative biological control of FAW under ecological conditions and agricultural systems in Africa. This study contributed to addressing this knowledge gap by conducting replicated field release trials of *T. remus* for FAW control in large maize plots in Ghana. In the major and minor rainy seasons, *T. remus* parasitized up to 33% and 100% of FAW egg masses, respectively, in the release plots. However, similar effectiveness of *T. remus* was recorded in non-treated control and farmers’ plots located at 150–400 m distance from release plots, indicating high dispersion of the parasitoid. A single application of Emamectin benzoate did not significantly affect the parasitism rates of *T. remus*, and could be considered in IPM strategies against FAW.

**Abstract:**

In response to the threat caused by the fall armyworm to African maize farmers, we conducted a series of field release studies with the egg parasitoid *Telenomus remus* in Ghana. Three releases of ≈15,000 individuals each were conducted in maize plots of 0.5 ha each in the major and minor rainy seasons of 2020, and compared to no-release control plots as well as to farmer-managed plots with chemical pest control. No egg mass parasitism was observed directly before the first field release. Egg mass parasitism reached 33% in the *T. remus* release plot in the major rainy season, while 72–100% of egg masses were parasitized in the minor rainy season, during which pest densities were much lower. However, no significant difference in egg mass parasitism was found among the *T. remus* release plots, the no-release control plots and the farmer-managed plots. Similarly, no significant decrease in larval numbers or plant damage was found in the *T. remus* release fields compared to the no-release plots, while lower leaf and tassel damage was observed in farmer-managed plots. Larval parasitism due to other parasitoids reached 18–42% in the major rainy season but was significantly lower in the minor rainy season, with no significant differences among treatments. We did not observe significant differences in cob damage or yield among the three treatments. However, the lack of any significant differences between the release and no-release plots, which may be attributed to parasitoid dispersal during the five weeks of observation, would require further studies to confirm. Interestingly, a single application of Emamectin benzoate did not significantly affect the parasitism rates of *T. remus* and, thus, merits further investigation in the context of developing IPM strategies against FAW.

## 1. Introduction

The fall armyworm (FAW), *Spodoptera frugiperda* (JE Smith) (Lepidoptera: Noctuidae), an invasive species originating from the tropical and subtropical regions of the Americas, was initially detected in 2016 in a few countries in West Africa, including Ghana. It has since spread rapidly throughout almost all sub-Saharan African countries, as well as many countries in Asia, and has also reached Oceania [1,2,3,4]. Maize is the main crop affected, which is of major concern as it contributes to the food security of more than 300 million households in Africa. The first measure adopted by governments of affected countries in Africa was often a distribution of free synthetic insecticides, even though this could have adverse effects on human and environmental health. Considering the large proportion of agricultural land used for maize cropping in most sub-Saharan African countries and the large number of rural workers involved, a continued reliance on chemicals is not sustainable. In addition to applying registered chemicals, farmers have also used unregistered ones, which are available on the market and are often cheaper [5]. Farmers often spray the same pesticide repeatedly, thereby increasing the risk of the development of insecticide resistance, as well as increasing production costs. There is, therefore, a need to develop and scale up IPM strategies against FAW, ideally with biological control as a key component.

A first approach to consider is classical biological control, due to its long-lasting effects and high level of cost-efficacy, if successful [6]. However, this approach may take considerable time to show results and there is also uncertainty as to whether the level of control would be adequate. Another approach is conservation biological control, in which the creation of suitable conditions to conserve natural enemies can play an important role in the control of pests such as FAW (e.g., [7]). Noctuids in general, including FAW, have high fecundity but at the same time are relatively easy prey for a large number of natural enemies, as shown in studies conducted in the Americas [8,9,10]. Interestingly, several natural enemies of FAW have been identified in Africa as early as 2017, including egg and larval parasitoids, and some of them already play a significant role in the control of the pest [11,12,13,14]. A third biological control approach is augmentative releases of natural enemies. For this, candidates occurring in the invaded area should be considered first, and exotic ones avoided, in order to minimise any potential non-target effects [15]. Among the natural enemies of FAW already reported in Africa, the egg parasitoid *Telenomus remus* (Nixon) (Hymenoptera: Platygastridae) looks particularly promising. *Telenomus remus* parasitizes various Lepidoptera species, mainly from the family Noctuidae, and especially *Spodoptera* spp. Originating from peninsular Malaysia, *T. remus* was introduced to various parts of the world for biological control of *Spodoptera* spp., including India, Pakistan, Australia, New Zealand, the Caribbean, Colombia and Venezuela [6]. The parasitoid is now found widely in the Americas but has recently also been found in several African countries, including Ghana [6]. A female produces an average of 270 eggs during her lifespan. It usually lays individually in each host egg, avoiding super parasitism. It is also able to parasitize the whole egg mass, whereas other egg parasitoids such as *Trichogramma* spp. tend to parasitize only part of the egg mass [16]. This is due to the fact that egg masses often consist of several layers and the female moth covers egg masses with scales from her body that provide a barrier to *Trichogramma* females, but not to *T. remus*. Studies from the Americas have shown that augmentative releases of *T. remus* in maize fields can result in high parasitism rates on sentinel egg masses of *S. frugiperda* [17,18,19] and such releases have been conducted on a large scale in Venezuela [20]. However, except one study that assessed the parasitism rate of *T. remus* on sentinel FAW egg masses in Africa [21], little is known about the real potential of *T. remus* to control FAW under ecological conditions and agricultural systems and practices in Africa. We aimed to address this knowledge gap by conducting replicated field release trials with *T. remus* for FAW control in large maize plots in Ghana. Due to possible dependencies and synergies, the parasitism rate from other local FAW parasitoids was also assessed.

## 2. Materials and Methods

### 2.1. Study Site

**Figure 1:**
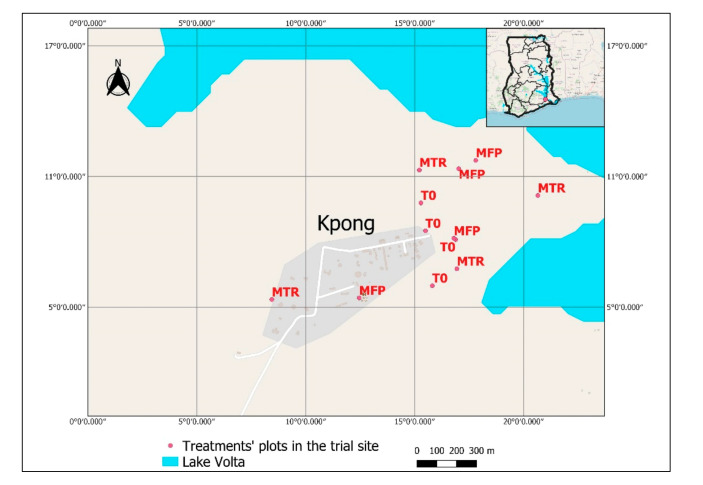
Study site indicating the position of plots for the three treatments: maize plots in which *T. remus* are released (MTR); maize plots with farmer practice (MFP); untreated control maize plots (T0).

The trial was conducted in 2020 at the Soil and Irrigation Research Centre (SIREC) of the University of Ghana and its environs, located in Kpong, Eastern region, Ghana (Figure 1). Kpong is located in the Coastal savanna agro-ecological zone of Ghana and is part of the Accra plains. It has an annual rainfall of between 700 and 1100 mm, an average annual temperature of 28 °C and a relative humidity of between 59 and 93% [22]. The rainfall distribution is bimodal with the possibility for farmers to have two growing seasons [23]. The Kpong area is crossed by the Volta river and non-agricultural areas are highly occupied by hills and gallery forests. The grassland in Kpong and its environs is dotted with trees such as mango, neem and Cassia, and annual crops including maize. The maize grown in the area has been heavily infested by FAW since its outbreak in Ghana. The soil type of the experimental site is heavy black clay known as the Vertisols, with a high water-holding capacity [24].

### 2.2. Experimental Design

The study was conducted using a completely randomized design to assess and compare the effectiveness of three treatments: *Telenomus* release (MTR), untreated control (T0), and farmers’ practice with pesticide application but no release of *Telenomus* (MFP). Each treatment was replicated 4 times in both the major and minor rainy seasons of 2020, with a minimum distance of 150 m between the release plots and any other plot. The space between the plots was occupied mostly by other crops (rice, tomato, onion), and different species of grasses and trees (including leguminous ones). Maize plots measured 0.5 ha for MTR and T0, but ranged between 0.4 and 0.5 ha for MFP.

### 2.3. General Crop Management

Crop management practices were implemented by staff of SIREC and were exactly the same in the MTR and the T0 treatments. In the farmer-managed MFP plots, a few practices were different, as explained below. A quality protein maize variety, Obatanpa, which is one of the most commonly planted maize varieties in the study area, was used on all plots. Maize was sown on 29 April and 30 September 2020 in the MTR and T0 plots, while sowing in the MFP plots was conducted a few days (maximum of 7 days) earlier or later. The planting layout was two maize plants per hole, 40 cm between holes in the row and 80 cm between rows. In the MTR and T0 treatments, the land was prepared using a tractor for tillage and weeds were managed by applying a pre-emergent herbicide (Agristomp 500E: Pendimethalin 500 g/L), followed by hoe weeding. In contrast, zero tillage was used in the MFP plots, and a pre-emergence herbicide (Sunphosate 360 SL: glyphosate 360 g/L) and a selective herbicide (Super Nicogan 800 WDG: 570 g/kg Maesotrione + 230 g/kg Nicosulfuron) were used for control of weeds.

To improve soil fertility, the fertilizer NPKS-20-10-10-3 was applied to the maize plants 10 days after planting at the rate of 200 kg/ha. Five weeks after planting, Urea (46%N) was applied at a rate of 100 kg/ha. In the MTR and T0 plots, each fertilizer was applied in small holes near individual maize plants and then covered by soil, while in the MFP plots it was dropped on the soil near the plants.

For FAW control, the farmers of the MFP plots applied an Emamectin benzoate-based product (Ataka Super^®^ EC: Emamectin benzoate 19.2 g/L), one of the most effective insecticides used by maize farmers in the area. During each of the two rainy seasons, farmers made only one insecticide application at the vegetative growth stage of the maize, after their own assessment of FAW infestation levels. No insecticide was applied against FAW or any other insect pests in the treatments MTR and T0.

### 2.4. Telenomus Remus Releases

Mass rearing of *T. remus* for release was conducted at the biocontrol laboratory of the Plant Protection and Regulatory Services Directorate (PPRSD), in Pokuase, Ghana. A culture was established using insects reared from parasitized FAW egg masses collected in maize fields in Somanya, Ghana, in 2019, and subsequently reared on egg masses from a FAW population kept in the laboratory at PPRSD since 2018. Rearing was performed at a temperature of 27 ± 1 °C and a relative humidity of 60 ± 10%. Under these laboratory conditions, the sex ratio of *T. remus* offspring is around 1:1.

**Figure 2:**
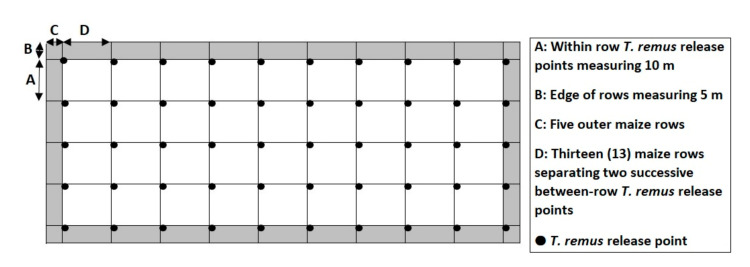
Scheme showing the 50 *Telenomus remus* release points in 0.5 ha maize plots with distances of 10 m within rows and 10.4 m between rows.

FAW larvae were reared on maize leaves that were replenished every two days. FAW adults emerged in aerated plastic jars of 0.75 L and were allowed to oviposit on maize leaves contained in cylindrical cages (r = 14.1 cm; h = 32 cm). FAW egg masses were collected daily from these cages, glued on a piece of white paper card, sterilized immediately in a UV light cabinet and stored in a refrigerator at 8 °C for a maximum of 7 days. Sterilized FAW egg masses were exposed to mated *T. remus* females (at the rate of 1.5 females per egg mass) in an oviposition cage (30 × 20 × 50 cm^3^) for 24 to 48 h for parasitization. Parasitized egg masses were kept in aerated transparent plastic jars until *T. remus* emergence, which started about 10 days after parasitization, with a mean emergence rate of 95.5%. For the field releases, FAW eggs, parasitized by *T. remus* in the laboratory and ready to emerge within 24 h, were taken to the field and released in the MTR plots as follows (Figure 2):

- Non-sealed letter envelopes containing approximately 300 parasitized eggs were fastened on maize plants (or on sticks when the maize plants were less than three weeks old). They were spaced every 13th maize row (13 × 0.8 m = 10.4 m), without considering the 5 outer rows at both sides of the plot;
- Within the maize row, the envelopes containing the ≈300 parasitized eggs were set up every 10 m, leaving 5 m at the ends of the rows.

**Figure 3:**
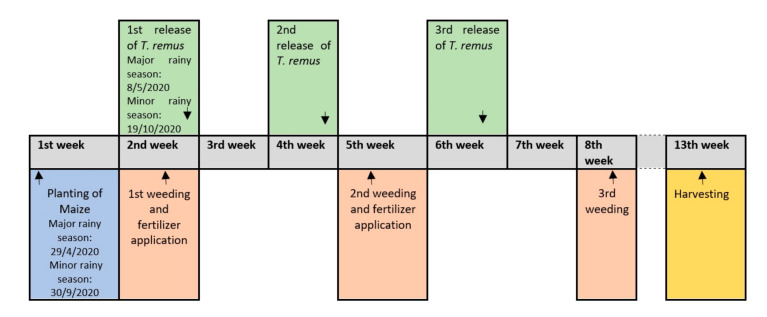
Overview on timelines for *Telenomus remus* releases and relevant crop management procedures in MTR and T0 maize plots.

A total of approximately 15,000 *T. remus* were thus released at 50 points in each 0.5 ha MTR plot and this was repeated three times during each of the two seasons (Figure 3). During the first trial conducted in the major rainy season from May to August 2020, the first release of *T. remus* was conducted 14 days after the maize was planted. The two subsequent releases of *T. remus* were performed biweekly thereafter. The trial was repeated in the minor rainy season, from September 2020 to January 2021, with the first *T. remus* released 20 days after planting the maize. The slight delay of the first release of *T. remus* during this minor rainy season trial was due to the high frequency of rains delaying the build-up of FAW infestations in the maize plots. As in the first trial, the second and third releases of *T. remus* were undertaken fortnightly after the first release.

### 2.5. Data Collection

The parameters assessed during this study were parasitism level of FAW by *T. remus* and other parasitoids, FAW infestation level, plant damage, tassel damage, cob damage, and maize yield. On each of the four plots of each treatment, data were collected on 75 plants per assessment within the plot, avoiding the edges, and following the “X” pattern (15 plants at the 5 locations (spots) on the “X” pattern). For both the major and the minor rainy season trials, the first field data on parasitism, FAW infestation level and plant damage were collected prior to the first release of *T. remus* on the same date, in order to serve as a reference. Subsequent field data were collected weekly until maize tasselling to assess egg parasitism, and biweekly to assess larval parasitism, FAW infestation level and plant damage.

All FAW egg masses and young larvae (1st, 2nd and 3rd instar) recorded from the 75 plants in each plot were collected and kept separately in aerated transparent plastic cups (V: 80 mL) containing a piece of tissue paper, and transferred to the laboratory, under ambient temperatures. Older FAW larvae (4th, 5th and 6th instar) were recorded without being removed from the plots. When very few or no FAW egg masses or young larvae were collected on the 75 maize plants sampled, extra egg masses or young larvae were collected immediately from other plants nearby in order to increase the number of eggs and larvae available for parasitism level assessment. All the *T. remus* individuals that emerged from the samples in the laboratory were conserved in 70% alcohol and their identity was confirmed based on morphological characters and comparison with barcoded voucher specimens deposited into GenBank [13]. Egg mass parasitism was calculated as the proportion of total egg masses collected that showed any parasitized eggs. The FAW larvae kept in the laboratory were provided with fresh untreated maize leaves every two days until the emergence of larval parasitoids or FAW adults. The larval parasitoids were identified by comparison with voucher specimens [13,14] and new species not included in the lists were identified by the author (MK). Larval parasitism was estimated as the proportion of FAW larvae collected in the plot that were parasitized. The relative abundance of larval parasitoids was the number of individuals of each parasitoid species as a percentage of the total number of larval parasitoids collected during the season.

Crop damage was assessed using the Davis scale, which goes from 0 (no damage) to 9 and combines both incidence and severity information [25]; it does not, however, consider damage on maize tassels and cobs. Therefore, we also recorded the proportion of maize tassels or cobs damaged by FAW on the 75 plants observed per plot.

For the yield estimation, maize cobs were harvested at maturity from four subplots measuring 50 m^2^ located within each maize plot away from the edges. The harvested cobs were dried until moisture content was between 12 and 14%, and then weighed. The summed weight of the four subsamples was multiplied by 50 to obtain the yield per hectare.

### 2.6. Statistical Analysis

Data on fall armyworm larval numbers, egg mass parasitism, larval parasitism and plant damage were analysed using linear mixed models to account for the repeated measures taken, which cannot be considered independent data; LSD tests were used for post-hoc separation of means among treatments. Data on tassel damage, cob damage and maize grain yield were averaged for each plot and analysed using two-way ANOVA to test for differences between treatments (followed by Tukey’s HSD post-hoc tests) and the two seasons of the study. Data were analysed using IBM SPSS Statistics 25.0.

## 3. Results

### 3.1. Parasitism Rate by T. remus during the First and Second Rainy Seasons

Directly before the first release of *T. remus* in the major rainy season, 19 FAW egg masses were collected from all experimental maize plots, without any sign of parasitism. From the 74 larvae collected before release in the major rainy season, three (4.05%) were found to be parasitized. In the minor rainy season, once again, no egg parasitism was observed on the 22 egg masses collected directly before the first release of *T. remus*. Parasitism of the 121 larvae found before release in the minor rainy season was 7.44%.

**Figure 4:**
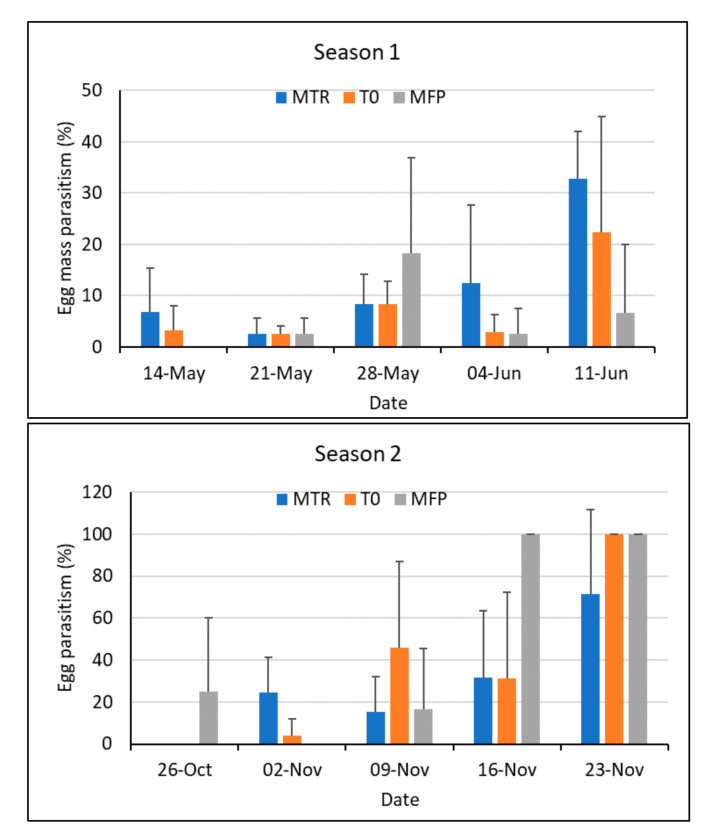
Mean (+SD) egg mass parasitism by *T. remus* in the major (**Season 1**) and minor (**Season 2**) rainy seasons in Ghana in 2020. MTR = *T. remus* release plots; T0 = plots without *T. remus* releases, no pest control; MFP = farmers’ conventional practice including chemical pest control; 4 replicates per treatment and season.

During the course of five evaluations made during the maize vegetative stage after parasitoid releases in the two rainy seasons, a total of 1424 egg masses were collected and assessed. From these, 188 egg masses were parasitized by *T. remus*, with a few Trichogramma wasps emerging together with *T. remus* from seven of the parasitized egg masses. Of the parasitized egg masses, 52.7% were fully parasitized and the remaining 47.3% were mostly parasitized. Overall, egg mass parasitism rates were not significantly different among treatments during the major rainy season (Linear Mixed Models, *F*_2, 12.046_ = 0.34, *p* = 0.72, Figure 4), during the minor rainy season (*F*_2, 17.429_ = 1.09, *p* = 0.36), or when looking at both seasons combined (*F*_2, 41.443_ = 1.27, *p* = 0.29, Figure 4).

Fewer FAW egg masses were collected in the minor rainy season (*n* = 211) than in the major rainy season (*n* = 1213). Egg parasitism was significantly higher during the minor rainy season than in the major rainy season (*F*_2, 41.193_ = 10.5, *p* = 0.002). However, the parasitism rate increased gradually after each release in the major rainy season, and similarly in the minor rainy season, even though here the pattern was not very consistent.

### 3.2. FAW Damage and Larval Density under Different Treatments

**Figure 5:**
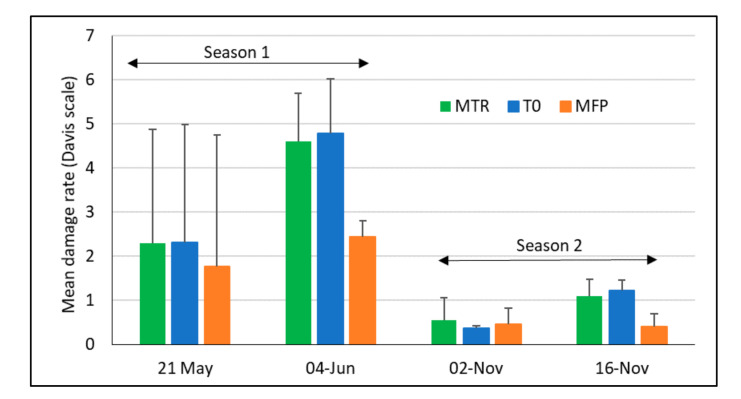
Mean plant damage (+SD) assessed on the Davis scale, during the two maize growing seasons in Ghana in 2020. MTR = *T. remus* release plots; T0 = plots without *T. remus* releases, no pest control; MFP = farmers’ conventional practice incl. chemical pest control; 4 replicates per treatment and season.

Plant damage assessed using the Davis scale was significantly different among treatments (*F*_2, 24.819_ = 8.01, *p* = 0.002, Figure 5). In both rainy seasons, damage increased with time in the MTR and T0 treatments but stayed relatively stable in the MFP treatment, leading to significant differences between MTR and MFP (*p* = 0.003) as well as between T0 and MFP (*p* = 0.001). Mean damage rates reached nearly five later in the major rainy season and stayed significantly lower in the minor rainy season (*F*_2, 24.860_ = 91.2, *p* < 0.001).

**Figure 6:**
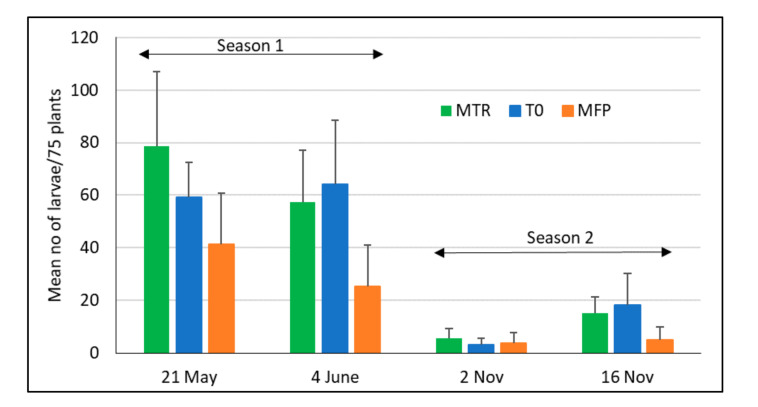
Mean (+SD) FAW larval numbers during the two maize growing seasons in Ghana in 2020. MTR = *T. remus* release plots; T0 = plots without *T. remus* releases, no pest control; MFP = farmers’ conventional practice including chemical pest control; 4 replicates per treatment and season.

The numbers of FAW were significantly different among treatments (*F*_2, 43.913_ = 6.90, *p* = 0.002, Figure 6) with significantly lower numbers in the farmers’ practice (MFP) compared to MTR (*p* = 0.001) and T0 (*p* = 0.005). Larval numbers were higher in the major rainy season compared to the minor rainy season (*F*_2, 43.913_ = 87.9, *p* = 0.002).

### 3.3. Seasonal Abundance of Larval Parasitoids and Their Parasitism Rate during the Trials

Several species of larval parasitoids and one species of egg–larval parasitoid were collected in the two seasons. The most commonly found species were Chelonus bifoveolatus Szépligeti (Hym.: Braconidae), Coccygidium luteum (Brullé) (Hym.: Braconidae) and Charops sp. (Hym.: Ichneumonidae) (Table 1). Mortality of collected larvae due to unknown reasons was 13% in both seasons.

**Figure 7:**
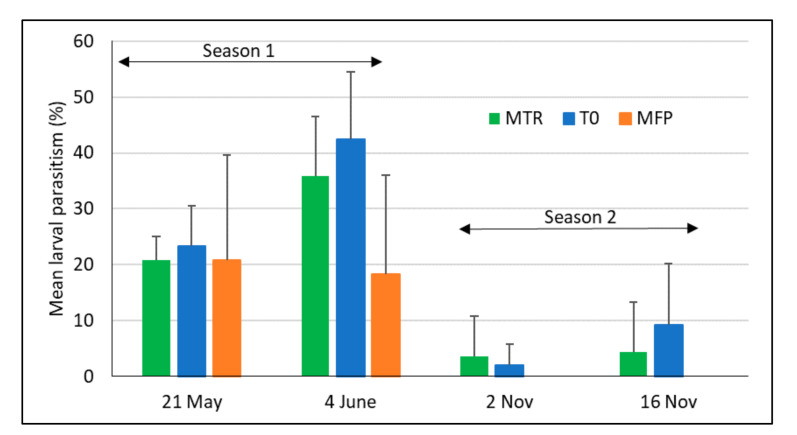
Mean parasitism of FAW larvae (+SD) during the two maize growing seasons in Ghana in 2020. MTR = *T. remus* release plots; T0 = plots without *T. remus* releases, no pest control; MFP = farmers’ conventional practice including chemical pest control; 4 replicates per treatment and season.

Generally, the combined parasitism of FAW larvae for all species was rather variable and, overall, significantly lower in the minor rainy season (*F*_2, 36.312_ = 49.3, *p* < 0.001, Figure 7). Larval parasitoids were able to cause up to 40% parasitism in the major rainy season but this parasitism level decreased in the minor rainy season for all the treatments. No parasitism was observed in the farmers’ practice treatment in the minor rainy season but combined larval parasitism during the two seasons did not show any significant differences among the three treatments (*F*_2, 36.233_ = 1.88, *p* = 0.17). There was a significant positive relationship between larval density and larval parasitism (r^2^ = 0.46, *F*_1, 43_ = 36.3, *p* < 0.001).

### 3.4. FAW Damage on Maize Tassels and Cobs, and Yield under Different Treatments

**Figure 8:**
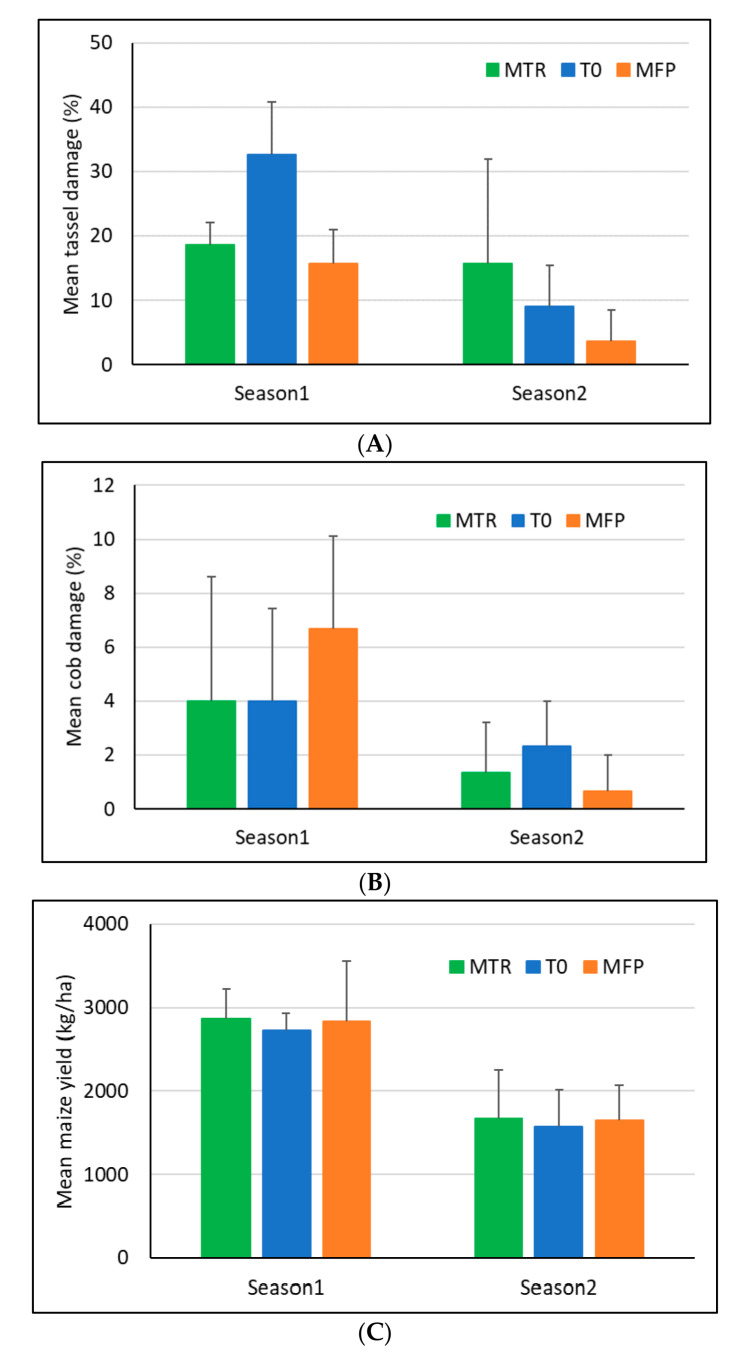
Mean (+SD) tassel damage (**A**), cob damage (**B**) and maize grain yield (**C**) during the two maize growing seasons in Ghana in 2020. MTR = *T. remus* release plots; T0 = plots without *T. remus* releases, no pest control; MFP = farmers’ conventional practice including chemical pest control; 4 replicates per treatment and season.

There were significant differences in the proportions of plants showing tassel damage among treatments (*F*_2, 18_ = 3.55, *p* = 0.05), with the pairwise comparison between T0 and MFP being the only significant one (*p* = 0.044; Figure 8A). When considering only the major rainy season where FAW infestation was higher, tassel damage was highly significantly different between treatments (*F*_1, 9_ = 9.17, *p* = 0.007), with significantly lower tassel damage in the MTR and MFP treatments (*p* = 0.023 and 0.008, respectively), compared to the no-release treatment T0. Significantly higher tassel damage was observed in the major rainy season as compared to the minor rainy season (*F*_1, 18_ = 13.64, *p* = 0.002).

No significant difference between treatments was found in the proportion of cobs damaged overall (*F*_2, 18_ = 0.23, *p* = 0.80, Figure 8). Significantly higher cob damage was observed in the major rainy season as compared to the minor rainy season (*F*_1, 18_ = 8.03, *p* = 0.011). Yield was rather variable within treatments and no significant difference was found among them (*F*_2, 18_ = 0.12, *p* = 0.89, Figure 8). Yield was highly significantly lower in the minor rainy season compared to the major rainy season (*F*_2, 18_ = 35.1, *p* < 0.001).

## 4. Discussion

Although *T. remus* is frequently cited as a promising augmentative biological control agent against FAW in Africa and Asia [6,26,27,28,29], few publications provide solid data to assess the efficacy of the method under natural infestation conditions. This is the first published study outside the native range of the pest describing trials in farm conditions and assessing the impact on naturally occurring FAW populations during two maize growing seasons. The authors of [21] conducted field trials in sorghum in Niger, but these were carried out in much smaller plots (200 m^2^) and parasitism was assessed on sterile eggs exposed in plots for four days only. From the few publications describing field tests in the Americas, nearly all used small experimental plots with sentinel eggs exposed close to the release points, which makes comparisons with our study very difficult.

At first view, our trials fail to provide evidence that releases were successful because no significant differences in egg mass parasitism were found between the treatment and the two controls. However, high or very high rates of parasitism were observed in the second half of the two seasons (over 30% and up to 100%, respectively) whereas no parasitized egg mass was found at the beginning of the experiments. This suggests that our releases may have had a general impact on parasitism during the season, and that the distance between the plots was not sufficiently large to prevent spread from the release plot to other nearby fields, particularly in the minor rainy season when host populations were low and egg masses were rare. All control plots were situated between 150 m and 400 m from release sites. The minimum distance of 150 m was established based on dispersal experiments stating that *T. remus* females do not spread more than a few dozen meters when released in maize fields [19,30]. However, those observations were made with high numbers of sentinel eggs exposed beside the released wasps, and for a maximum of four days after the releases. It is likely that, when eggs are less abundant, females will fly longer distances. Furthermore, in this experiment, *T. remus* had the possibility of passing through three generations in the five weeks of observation after the releases, increasing the dispersal potential. In the releases of *T. remus* in Venezuela, parasitism rates of over 70% were observed 2 km away from the release site just two months after the releases [31]. Opportunistic egg mass collections in July 2019 and July 2020, 5–7 km from our experimental sites, showed that *T. remus* is naturally present in the area [13]. While it is unfortunate that no survey was made during the trials in 2020 to quantitatively assess natural egg parasitism in the area around our experimental site, and in areas sufficiently far from the release sites, we consider it unlikely that *T. remus* egg mass parasitism could naturally reach 70–100% as observed in some of our control plots (T0 and MFP) without the releases made in the nearby plots (MTR). When conducting similar trials, we therefore strongly recommended that experimental fields are separated by several kilometres to avoid possible interference, especially when the effects of releases on parasitism rates are measured over several weeks.

The fact that no egg parasitism was found at the beginning of the two seasons suggests that *T. remus* does not survive well during the dry seasons. There was only two months of drought (August and September) between the two rainy seasons, but it may be sufficient to knock down *T. remus* populations either because they do not withstand drought or because too few hosts are available during the dry season. The fact that the offspring of *T. remus* released in the major rainy season did not lead to a persistent population in situ could also be due to the maize monocropping system not providing sufficient food resources for adult *T. remus*. The authors of [32] reported that such monocropping systems are considered as an ephemeral annual system where the intensity of disturbance is high and the quality of the environment is unsuitable for the establishment of released natural enemies, thereby constraining their success in controlling the target pest.

In the major rainy season, mean egg parasitism rates were low compared to other studies [18,19,21], but those studies used sentinel egg masses to measure parasitism. Sentinel eggs are often sterilized and, thus, available for parasitism for longer than normal eggs, which hatch after only 3 days under tropical conditions. It is noteworthy that Figueiredo et al., the authors of [18], found low parasitism rates on the naturally laid egg masses collected in that study. Parasitism rates are also related to the number of parasitoids released and to host density. In the present study, the number of *T. remus* released was ≈30,000 per ha, which is relatively low compared to other field studies and guidelines [18,19,21,28,33] that recommend 50,000 to 200,000 adults per ha. However, in Venezuela, which is the only country where *T. remus* has been used for biological control of fall armyworm continuously for many years, release rates have been much lower [20] and, nowadays, a release rate of 5000 wasps per ha is recommended (F. Ferrer, personal communication). Release rates should also be seen in the context of the fitness of released wasps, which may be related to the host they were reared on. Here, and in most other studies, *T. remus* was reared on *Spodoptera* eggs, implying relatively high production costs but, on the other hand, high parasitoid fitness, i.e., high fecundity, longevity and dispersal capacities [34,35]. In comparison, the widely used *Trichogramma* egg parasitoid is usually released in numbers of around 100,000 per release, but is often reared on small eggs, such as *Ephestia* sp (Lep.: Pyralidae), *Corcyra* sp (Lep.: Pyralidae) or *Sitotroga* sp (Lep.: Gelechiidae), resulting in small wasps. What the best release rate would be for *T. remus* is still to be determined, but 50,000–200,000 adults/ha is very unlikely to be economically sustainable. So far, efforts to develop a technology to mass rear *T. remus* on factitious hosts, such as the stored product moths *Corcyra cephalonica* (Stainton) and *Ephestia kuehniella* (Zeller), have unfortunately failed with the African *T. remus* strain.

Egg–larval and larval parasitoids were abundant. Up to 40% of FAW young larvae were found to be parasitized. This high parasitism rate is likely to represent a correct estimation of parasitoids developing in young larvae. In most other studies, larval parasitism rates were underestimated because a part of the larvae were collected in their last instar, when these parasitoids had already killed their host (e.g., [11,13,36,37]). In contrast, in this study focusing on the collection of younger larvae, the parasitism rates of the guild attacking younger larvae should be more accurate, while parasitoids attacking older larvae, such as tachinid flies, were missed. Larval parasitism was much lower in the minor rainy season than in the major one. This may be because the short rainy season is less favourable for the parasitoids or their native hosts. It may also be because the parasitoids are density-dependant, as shown in the same system in Zambia [14], and host populations were too low to be attractive for larval parasitoids due to the high egg parasitism in the minor rainy season.

The abundance of the egg–larval parasitoid *Ch. bifoveolatus* is particularly remarkable. Since it is an egg–larval parasitoid, it directly competes with egg parasitoids. It was shown that, in case of multiparasitism between *T. remus* and the American *Ch. insularis*, competition was in favour of *T. remus* [38], and it is likely that the same occurs with *Ch. bifoveolatus*.

The farmer practice tested in this study was based on one application of emamectin benzoate around one month after maize sowing. This single treatment with an insecticide of relatively low non-target toxicity [39] did not significantly affect *T. remus* and the larval parasitoids even though there was a tendency to lower larval parasitism in the farmers’ plots. Many farmers in Africa use more toxic insecticides and/or more frequent applications [40], which is likely to have an effect on natural enemies [41]. This apparent innocuity of one application of emamectin benzoate on FAW parasitoids could be considered when developing IPM strategies against the pest, and it opens perspectives for the use of *T. remus* or other parasitoids in IPM programmes. It is also interesting to note that while pesticide application had a significant effect on plant damage as well as on larval density when compared to the control plots, the effect on yields became insignificant. This resilience of maize to FAW damage is commonly observed when the seasonal rainfall is well distributed and good agronomic practices (e.g., weeding and fertilizer application) are adopted, and should be considered in decision-making processes in IPM systems [42,43,44].

## 5. Conclusions

This study showed that repeated releases of *T. remus* in maize fields were followed by increases in egg parasitism, but the effect was similar in the farmers’ practice fields and control fields, possibly because *T. remus* dispersed further than expected during the five weeks of observation. New trials should be conducted with control plots situated much further from the release plots, ideally including monitoring of the occurrence of *T. remus* over a larger area and longer time periods to better understand dispersal capacities of this egg parasitoid. If it is confirmed that *T. remus* can disperse rapidly, its use in biological control programmes could be considered following an area-wide management approach, or even based on an annual or biannual inoculative approach. High dispersal would also indicate that the number of release points could be drastically reduced, making releases easier and cheaper. Interestingly, egg–larval and larval parasitoids were particularly abundant at high host density and one application of Emamectin benzoate did not affect parasitism rates significantly, offering a possibility to consider the use of *T. remus* and potentially other parasitoids within IPM strategies.

## Figures and Tables

**Table 1:** Parasitoid species emerged from Spodoptera frugiperda larvae and their relative abundance during the major and minor rainy seasons in 2020.

Season	Species (Order: Family)	Host Stage Attacked/Killed	Relative Abundance (%) * *n*_1_ = 184; ** *n*_2_ = 22
Major rainy season	*Chelonus bifoveolatus* Szépligeti (Hym.: Braconidae)	Egg–larval	63.04
	*Chelonus* cf. *curvimaculatus* Cameron		0.54
	*Coccygidium luteum* (Brullé) (Hym.: Braconidae)	Larval	20.65
	*Charops* sp. (Hym.: Ichneumonidae)		5.98
	*Cotesia icipe* Fernandez-Triana and Fiaboe (Hym.: Braconidae)	Larval	3.26
	Undetermined specimens	Larval	7.07
Minor rainy season	*Chelonus bifoveolatus*	Egg–larval	31.82
	*Chelonus* cf. *curvimaculatus*	Egg–larval	9.09
	*Coccygidium luteum*	Larval	22.73
	*Charops* sp.	Larval	31.82
	Undetermined specimens	Larval	4.55

* *n*_1_: Total number of larval parasitoids of FAW during the major rainy season; ** *n*_2_: Total number of larval parasitoids of FAW during the minor rainy season.

## Data Availability

Not applicable.

## References

[1] Goergen G., Kumar P.L., Sankung S.B., Togola A., Tamò M. (2016). First report of outbreaks of the fall armyworm Spodoptera frugiperda (J E Smith) (Lepidoptera, Noctuidae), a new alien invasive pest in west and central africa. PLoS ONE.

[2] https://www.agric.wa.gov.au/fall-armyworm-western-australia.

[3] Day R., Abrahams P., Bateman M., Beale T., Clottey V., Cock M., Colmenarez Y., Corniani N., Early R., Godwin J. (2017). Fall Armyworm: Impacts and Implications for Africa. Outlooks Pest Manag..

[4] Nagoshi R.N., Ni Htain N., Boughton D., Zhang L., Xiao Y., Nagoshi B.Y., Mota-Sanchez D. (2020). Southeastern Asia fall armyworms are closely related to populations in Africa and India, consistent with common origin and recent migration. Sci. Rep..

[5] Kansiime M.K., Mugambi I., Rwomushana I., Nunda W., Lamontagne-Godwin J., Rware H., Phiri N.A., Chipabika G., Ndlovu M., Day R. (2019). Farmer perception of fall armyworm (*Spodoptera frugiderda* J.E. Smith) and farm-level management practices in Zambia. Pest Manag. Sci..

[6] Kenis M., Du Plessis H., Berg J.V.D., Ba M.N., Goergen G., Kwadjo K.E., Baoua I., Tefera T., Buddie A., Cafà G. (2019). Telenomus remus, a candidate parasitoid for the biological control of Spodoptera frugiperda in Africa, is already Present on the Continent. Insects.

[7] Harrison R.D., Thierfelder C., Baudron F., Chinwada P., Midega C., Schaffner U., Berg J.V.D. (2019). Agro-ecological options for fall armyworm (*Spodoptera frugiperda* JE Smith) management: Providing low-cost, smallholder friendly solutions to an invasive pest. J. Environ. Manag..

[8] Murúa M.G., Molina-Ochoa J., Fidalgo P. (2009). Natural Distribution of Parasitoids of Larvae of the Fall Armyworm, Spodoptera frugiperda, in Argentina. J. Insect Sci..

[9] Rios-Velasco C., Gallegos-Morales G., Cambero-Campos J., Cerna-Chávez E., Del Rincón-Castro M.C., Valenzuela-García R. (2011). Natural Enemies of the Fall Armyworm Spodoptera frugiperda (Lepidoptera: Noctuidae) in Coahuila, México. Fla. Èntomol..

[10] Wang W., He P., Zhang Y., Liu T., Jing X., Zhang S. (2020). The Population Growth of Spodoptera frugiperda on Six Cash Crop Species and Implications for Its Occurrence and Damage Potential in China. Insects.

[11] Sisay B., Simiyu J., Mendesil E., Likhayo P., Ayalew G., Mohamed S., Subramanian S., Tefera T. (2019). Fall Armyworm, Spodoptera frugiperda Infestations in East Africa: Assessment of Damage and Parasitism. Insects.

[12] Agboyi L.K., Goergen G., Beseh P., Mensah S.A., Clottey V.A., Glikpo R., Buddie A., Cafà G., Offord L., Day R. (2020). Parasitoid Complex of Fall Armyworm, Spodoptera frugiperda, in Ghana and Benin. Insects.

[13] Agboyi L.K., Mensah S.A., Clottey V.A., Beseh P., Glikpo R., Rwomushana I., Day R., Kenis M. (2019). Evidence of Leaf Consumption Rate Decrease in Fall Armyworm, Spodoptera frugiperda, Larvae Parasitized by Coccygidium luteum. Insects.

[14] Durocher-Granger L., Mfune T., Musesha M., Lowry A., Reynolds K., Buddie A., Cafà G., Offord L., Chipabika G., Dicke M. (2020). Factors influencing the occurrence of fall armyworm parasitoids in Zambia. J. Pest Sci..

[15] De Clercq P., Mason P.G., Babendreier D. (2011). Benefits and risks of exotic biological control agents. BioControl.

[16] Cave R.D. (2000). Biology, ecology and use in pest management of Telenomus remus. Biocontrol News Inf..

[17] Gutierrez-Martinez A., Tolón-Becerra A., Lastra-Bravo X.B. (2012). Biological control of spodoptera frugiperda eggs using Telenomus remus nixon in maize-bean-squash polyculture. Am. J. Agric. Biol. Sci..

[18] Figueiredo M., Lucia T., Cruz I. (2002). Effect of Telenomus remus Nixon (Hymenoptera: Scelionidae) Density on Control of Spodoptera frugiperda (Smith) (Lepidoptera: Noctuidae) egg masses upon release in a maize Field. Rev. Bras. Milho Sorgo.

[19] Salazar-Mendoza P., Rodriguez-Saona C., Fernandes O.A. (2020). Release density, dispersal capacity, and optimal rearing conditions for Telenomus remus, an egg parasitoid of Spodoptera frugiperda, in maize. Biocontrol Sci. Technol..

[20] Ferrer F. (2001). Biological control of agricultural insect pests in Venezuela; advances, achievements, and future perspectives. Biocontrol News Inf..

[21] Laminou S.A., Ba M.N., Karimoune L., Doumma A., Muniappan R. (2020). Parasitism of locally recruited egg parasitoids of the fall armyworm in Africa. Insects.

[22] Fening K.O., Forchibe E.E., Afreh-Nuamah K. (2020). Neem as a cost-effective and potent biopesticide against the diamondback moth *Plutella xylostella* L. (Lepidoptera: Plutellidae) and the cabbage webworm *Hellula undalis* F. (Lepidoptera: Crambidae). West Afr. J. Appl. Ecol..

[23] Nkrumah F., Klutse N.A.B., Adukpo D.C., Owusu K., Quagraine K.A., Owusu A., Gutowski W. (2014). Rainfall variability over Ghana: Model versus rain gauge observation. Int. J. Geosci..

[24] Fening K.O., MacCarthy D.S., Tegbe R.E. (2020). The Effect of intercropping and soil amendment on the population dynamics of pests and natural enemies of white cabbage. West Afr. J. Appl. Ecol..

[25] Davis F.M., Ng S.S., Williams W.P. (1992). Visual rating scales for screening whorl-stage corn for resistance to fall armyworm. Miss Agric. For. Exp. Stn. Tech. Bull..

[26] Prasanna B.M., Huesing J.E., Eddy R., Peschke V.M. (2018). Fall Armyworm in Africa: A Guide for Integrated Pest Management.

[27] Liao Y.-L., Yang B., Xu M.-F., Lin W., Wang D.-S., Chen K.-W., Chen H.-Y. (2019). First report of Telenomus remus parasitizing Spodoptera frugiperda and its field parasitism in southern China. J. Hymenopt. Res..

[28] Tefera T., Goftishu M., Ba M., Muniappan R. (2019). A Guide to Biological Control of Fall Armyworm in Africa Using Egg Parasitoids.

[29] Elibariki N., Bajracharya A.S.R., Bhat B., Tefera T., Mottern J.L., Evans G., Muniappan R., Yubak D.G., Pallangyo B., Likhayo P. (2020). Candidates for augmentative biological control of Spodoptera frugiperda (J E smith) in Kenya, Tanzania and Nepal. Indian J. Èntomol..

[30] Pomari-Fernandes A., Bueno A.D.F., De Bortoli S.A., Favetti B.M. (2018). Dispersal capacity of the egg parasitoid Telenomus remus Nixon (Hymenoptera: Platygastridae) in maize and soybean crops. Biol. Control.

[31] Hernández D., Ferrer F., Linares B. (1989). Introducion de *Telenomus remus* Nixon (Hym.: Scelionidae) para controlar *Spodoptera frugiperda* (Lep.: Noctuidae) en Yaritagua-Venezuela. Agron. Trop..

[32] Landis D.A., Wratten S., Gurr G. (2000). Habitat Management to Conserve Natural Enemies of Arthropod Pests in Agriculture. Annu. Rev. Èntomol..

[33] Figueiredo M.L.C., Cruz I., Della Lucia T.M.C. (1999). Controle integrado de Spodoptera frugiperda (Smith and Abbott) utilizando-se o parasitóide Telenomus remus Nixon. Pesqui. Agropecu. Bras..

[34] Gao S., Tang Y., Wei K., Wang X., Yang Z., Zhang Y. (2016). Relationships between Body Size and Parasitic Fitness and Offspring Performance of Sclerodermus pupariae Yang et Yao (Hymenoptera: Bethylidae). PLoS ONE.

[35] West S., Flanagan K., Godfray H. (1996). The Relationship between Parasitoid Size and Fitness in the Field, a Study of Achrysocharoides zwoelferi (Hymenoptera: Eulophidae). J. Anim. Ecol..

[36] Sisay B., Simiyu J., Malusi P., Likhayo P., Mendesil E., Elibariki N., Wakgari M., Ayalew G., Tefera T. (2018). First report of the fall armyworm, Spodoptera frugiperda (Lepidoptera: Noctuidae), natural enemies from Africa. J. Appl. Èntomol..

[37] Otim M., Aropet S.A., Opio M., Kanyesigye D., Opolot H.N., Tay W.T. (2021). Parasitoid Distribution and Parasitism of the Fall Armyworm Spodoptera frugiperda (Lepidoptera: Noctuidae) in Different Maize Producing Regions of Uganda. Insects.

[38] Earl S.L. (1983). Competitive interactions between Chelonus insularis Cresson and Telenomus remus Nixon, Two Parasitoids of Spodoptera exigua Hubner. Master’s Thesis.

[39] Liu Y., Li X., Zhou C., Liu F., Mu W. (2016). Toxicity of nine insecticides on four natural enemies of Spodoptera exigua. Sci. Rep..

[40] Tambo J.A., Day R.K., Lamontagne-Godwin J., Silvestri S., Beseh P.K., Oppong-Mensah B., Phiri N.A., Matimelo M. (2020). Tackling fall armyworm (Spodoptera frugiperda) outbreak in Africa: An analysis of farmers’ control actions. Int. J. Pest Manag..

[41] Tang S., Tang G., Cheke R.A. (2010). Optimum timing for integrated pest management: Modelling rates of pesticide application and natural enemy releases. J. Theor. Biol..

[42] Baudron F., Zaman-Allah M.A., Chaipa I., Chari N., Chinwada P. (2019). Understanding the factors influencing fall armyworm (*Spodoptera frugiperda* J.E. Smith) damage in African smallholder maize fields and quantifying its impact on yield. A case study in Eastern Zimbabwe. Crop Prot..

[43] Kumar R., Singh D.P., Tiwana U.S. (2020). Forage yield compensation in maize with differential seed rates against insect herbivory of *Chilo partellus* (Swinhoe.). Range Manag. Agrofor..

[44] Liu G., Yang Y., Liu W., Guo X., Xue J., Xie R., Ming B., Wang K., Hou P., Li S. (2020). Leaf Removal Affects Maize Morphology and Grain Yield. Agronomy.

